# Azimuth Sidelobes Suppression Using Multi-Azimuth Angle Synthetic Aperture Radar Images

**DOI:** 10.3390/s19122764

**Published:** 2019-06-19

**Authors:** Yamin Wang, Wei Yang, Jie Chen, Hui Kuang, Wei Liu, Chunsheng Li

**Affiliations:** 1 School of Electronic and Information Engineering, Beihang University, Beijing 100191, China; wangyamin@buaa.edu.cn (Y.W.); yangweigigi@sina.com (W.Y.); kuanghui@buaa.edu.cn (H.K.); lics@buaa.edu.cn (C.L.); 2 Electronic and Electrical Engineering Department, University of Sheffield, Sheffield S1 3JD, UK; w.liu@sheffield.ac.uk

**Keywords:** multi-pass squinted (MPS), azimuth sidelobes suppression, synthetic aperture radar (SAR)

## Abstract

A novel method is proposed for azimuth sidelobes suppression using multi-pass squinted (MPS) synthetic aperture radar (SAR) data. For MPS SAR, the radar observes the scene with different squint angles and heights on each pass. The MPS SAR mode acquisition geometry is given first. Then, 2D signals are focused and the images are registered to the master image. Based on the new signal model, elevation processing and incoherent addition are introduced in detail, which are the main parts for azimuth sidelobes suppression. Moreover, parameter design criteria in incoherent addition are derived for the best performance. With the proposed parameter optimization step, the new method has a prominent azimuth sidelobes suppression effect with a slightly better azimuth resolution, as verified by experimental results on both simulated point targets and TerraSAR-X data.

## 1. Introduction

For many applications of synthetic aperture radar (SAR), the images can be adversely affected by sidelobes, especially in the case of strongly scattering targets with weak targets nearby, such as in a harbour with ships and containers. Hence, it is often desirable to suppress the sidelobes in order to improve image quality.

Several methods have been proposed to do this. The most common approach [1,2,3] imposes a weighting on the signal spectrum, such as the Taylor and Hamming windows, but such methods tend to widen the mainlobe. Another method, known as spatially variant apodization (SVA) [4,5], and its modified versions [6,7,8,9], can reduce the sidelobes without degrading mainlobe resolution. However, nonlinear apodization modifies the statistical distribution of the pixel intensities, thus hindering the extraction of information from homogeneous regions [10]. In [11], a dual-Delta factorization method was proposed to suppress sidelobes in squinted and bistatic SAR images, but this iterative method is complex and computationally expensive.

This letter introduces a novel multi-pass squinted (MPS) SAR, whose data can be used to realize 3D imaging and also to produce 2D images with low azimuth sidelobes. Compared with traditional multi-pass SAR for 3D imaging [12] or multi-baseline SAR for interferometry [13], in which the SAR operates in broadside mode, MPS SAR works in squint mode and observes the scene with different azimuth squint angles on each pass. Squint mode increases the difficulty of SAR signal processing and can also provide more possibilities in terms of applications with corresponding imaging methods. In this paper it is a novel application for azimuth sidelobes suppression based on MPS SAR mode; it can suppress azimuth sidelobes significantly and improve the azimuth resolution slightly simultaneously. For azimuth sidelobes suppression, the first-order phase related to the Doppler centroid frequency is specially preserved, which can be utilized in elevation processing. Some existing algorithms have been adjusted to cater for this new signal model in elevation processing, resulting in the azimuth mainlobe and sidelobes separating in elevation. Moreover, through the parameter optimization of elevation integrated range, the performance of the azimuth sidelobes suppression can be better, which is firstly introduced based on MPS SAR mode. The effectiveness of the proposed method is verified by both simulated data and the real TerraSAR-X image data compared with the signal spectrum weighting algorithms.

This letter describes a method for azimuth sidelobes suppression using MPS SAR data. In Section 2, the imaging geometry is introduced. Section 3 builds the signal model and describes the processing of the stack of images along elevation to yield resolution cells at different elevations, as in tomography. However, unlike tomography, in this mode the azimuth sidelobes occur at different elevations and can be eliminated by incoherent addition in elevation. The performance of the proposed method is related to system parameters in Section 4, and a set of design criteria is proposed. Simulations with point targets and real SAR images are performed to test the proposed method in Section 5 and conclusions are drawn in Section 6.

## 2. Multi-Pass Squinted SAR

**Figure 1:**
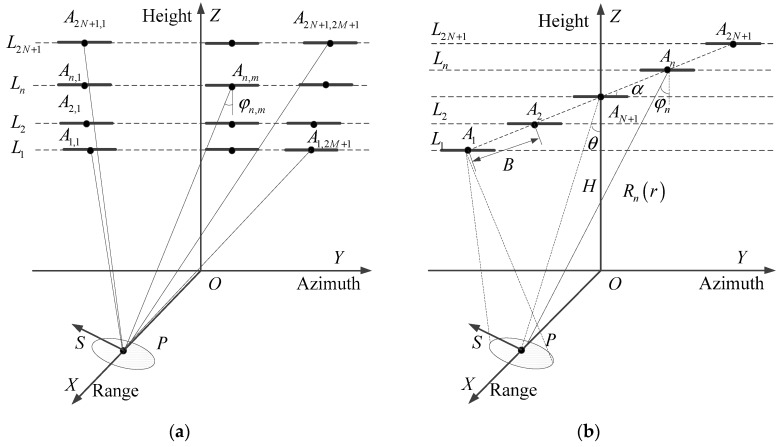
Imaging geometry: (**a**) MPS SAR; (**b**) for azimuth sidelobes suppression.

The imaging geometry of MPS SAR is shown in Figure 1a, where *X*, *Y*, *Z*, and S represent range, azimuth, height, and elevation coordinates, respectively. The aircraft is in the azimuth-height plane, $L_{n}$ represents the *n*th pass of the aircraft, $A_{n,m}$ is the center position of the SAR in the *m*th acquisition on the *n*th pass, 2N+1 and 2M+1 are the numbers of passes and acquisitions, respectively, $\varphi_{n,m}$ is the azimuth squint angle, which is the angle between line-of-sight and broadside, and the heavy lines on each pass represent the synthetic apertures of the acquisitions.

Based on the imaging geometry in Figure 1a, data acquired with the same azimuth squint angle on each pass can be combined and processed for 3D imaging, which is similar to the traditional TomoSAR. Moreover, the data acquired with different azimuth squint angles on each pass can be selected and used to suppress azimuth sidelobes. However, the acquisition of the data should meet the imaging geometry for azimuth sidelobes suppression, as shown in Figure 1b. Figure 1b represents the 2N+1 MPS acquisitions taken from each pass in Figure 1a; $A_{n}$ is the center position of the acquisition on the *n*th pass, $\varphi_{n}$ is the azimuth squint angle, H is the height of the center pass, θ is the incidence angle and α is the “flight angle”, which is the angle between the line of the center positions (assumed collinear and equally spaced) and the azimuth coordinate; this is given by $\alpha =\mathrm{acos}\left(\left(\vec{A_{1}A_{n}}\cdot \vec{y}\right)/\left|\vec{A_{1}A_{n}}\right|\right)$. $B=\left|\vec{A_{n}A_{n+1}}\right|$ is the distance between two adjacent center positions of the SAR and is referred to as the baseline (assumed to be the same for all adjacent pairs), and $B_{a,n}$, $B_{//,n}$, and $B_{⊥,n}$ are the azimuth, parallel, and orthogonal baselines, respectively, which are the projections of the vector $\vec{A_{n}A_{N+1}}$ along the azimuth, the line of sight, and elevation, with the following forms:

(1) $$\{\begin{array}{l} B_{a,n}=\left(n-N-1\right)\cdot B\cdot \cos\alpha \\ B_{⊥,n}=\left(n-N-1\right)\cdot B\cdot \sin\alpha \cdot \sin\theta \\ B_{//,n}=\left(n-N-1\right)\cdot B\cdot \sin\alpha \cdot \cos\theta \end{array},$$

P is a point target in the scene, and  represents the distance between the SAR center position $A_{n}$ and P:

(2) $$R_{n}\left(r\right)\approx \sqrt{{\left(r+B_{//,n}\right)}^{2}+B_{⊥,n}^{2}+B_{a,n}^{2}}\approx \sqrt{{\left(r+B_{//,n}\right)}^{2}+{\left(B_{a,n}\right)}^{2}}+B_{⊥,n}^{2}/\left(2\sqrt{{\left(r+B_{//,n}\right)}^{2}+{\left(B_{a,n}\right)}^{2}}\right)$$

where *r* represents the distance between $A_{N+1}$ and P.

## 3. Signal Processing

**Figure 2:**
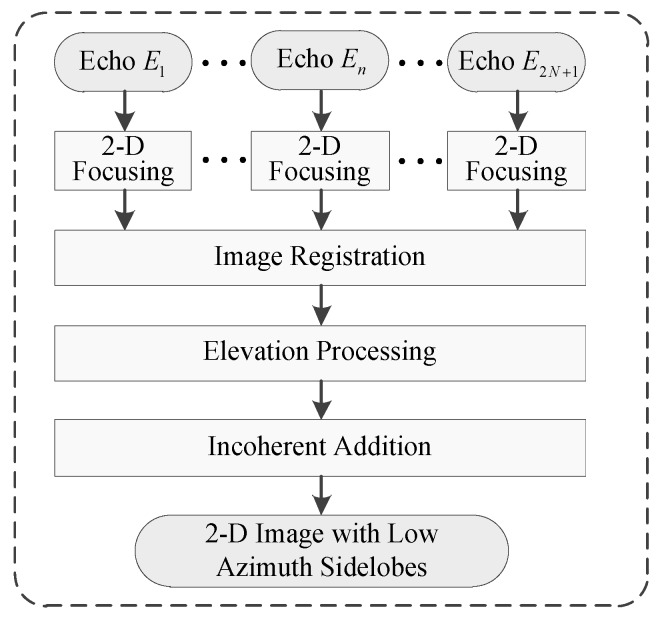
Flowchart of the proposed processing method for azimuth sidelobes suppression.

The signal model was built and the proposed method for suppressing azimuth sidelobes was derived based on the imaging geometry described in Section 2. It contained four main steps: 2D focusing, image registration, elevation processing, and incoherent addition, as indicated in Figure 2.

The data acquired on each pass were first focused to obtain a 2D image. Here, the modified chirp scaling kernel [14], which is suitable for squinted SAR, was used to process the raw data to get focused 2D images. However, not only the energy was focused in this 2D image processing, but also the phase was preserved, which can be utilized in subsequent elevation processing for azimuth sidelobes suppression in the proposed method. The 2D images were then registered to the selected master image (n=N+1). This allowed the stack of images to be processed along elevation, where the 2D signal from the target P in the *n*th image is given by:

(3) $$s_{n}\left(y^{\prime },r^{\prime }\right)=\sqrt{\sigma_{p}}\cdot \sin c\left(\frac{r^{\prime }-r}{\rho_{r}}\right)\cdot \sin c\left(\frac{y^{\prime }}{\rho_{a}}\right)\cdot \exp\left\{-j\frac{4\pi }{\lambda }R_{n}\left(r\right)\right\}\;\cdot \exp\left\{-j2\pi f_{d,n}y^{\prime }/v\right\},$$

where $\sigma_{P}$ is the radar cross-section (RCS) of P, λ is wavelength, $\rho_{a}$ and $\rho_{r}$ represent azimuth and range resolution, respectively, $r^{\prime }$ and $y^{\prime }$ are the variables associated with the range and azimuth position of the focused image, v is the velocity of the SAR, and $f_{d,n}$ is the Doppler centroid frequency of the *n*th acquisition:

(4) $$f_{d,n}=2v\sin\varphi_{n}/\lambda \approx 2vB_{a,n}/\left(\lambda \sqrt{{\left(r+B_{//,n}\right)}^{2}+B_{a,n}^{2}}\right).$$

In Equation (3), the second exponential term is the specially preserved phase, which indicates that in different acquisitions the phase varies with the Doppler centroid frequency and the target’s azimuth position. The 2D focusing and image registration are the basics of the proposed method.

We assumed that the variation in the heights of the targets in the scene was less than the resolution in elevation (see Equation (9)), so the imaging area could be seen as effectively flat. Thus, the targets in the same 2D image cell cannot be separated in elevation after elevation processing.

As a new application, the spectral analysis (SPECAN) [15,16] algorithm was used to process the MPS SAR signal in elevation. The residual phase induced by varying center distance $R_{n}\left(r\right)$ was first compensated by multiplying the signal with its complex conjugate phase:

(5) $$H\left(r,n\right)=\exp\left\{j\frac{4\pi }{\lambda }R_{n}\left(r\right)\right\}\approx \exp\left\{j\frac{4\pi }{\lambda }\left(\sqrt{{\left(r+B_{//,n}\right)}^{2}+B_{a,n}^{2}}+B_{⊥,n}^{2}/\left(2\sqrt{{\left(r+B_{//,n}\right)}^{2}+{\left(B_{a,n}\right)}^{2}}\right)\right)\right\}.$$

After multiplication, the signal was modeled as:

(6) $$s_{n}{}^{\prime }\left(y^{\prime },r^{\prime }\right)=\sqrt{\sigma_{p}}\cdot \sin c\left(\frac{r^{\prime }-r}{\rho_{r}}\right)\cdot \sin c\left(\frac{y^{\prime }}{\rho_{a}}\right)\cdot \exp\left\{-j2\pi f_{d,n}\frac{y^{\prime }}{v}\right\},$$

Then, a new variable $\xi_{n}$ (different from that in [15,16]) was designed to focus the signal in elevation by the single Fourier transform (FFT), to give:

(7) $$ss\left(y^{\prime },r^{\prime },s\right)=\sum_{n=1}^{2N+1}\left(s_{n}{}^{\prime }\left(y^{\prime },r^{\prime }\right)\cdot \exp\left\{-j2\pi \xi_{n}s\right\}\right)\approx \left(2N+1\right)\sqrt{\sigma_{p}}\cdot \sin c\left(\frac{r^{\prime }-r}{\rho_{r}}\right)\cdot \sin c\left(\frac{y^{\prime }}{\rho_{a}}\right)\cdot \sin c\left(\frac{s+y^{\prime }\cot\alpha /\sin\theta }{\rho_{e}}\right)$$

where,

(8) $$\xi_{n}=2B_{⊥,n}/\left(\lambda \sqrt{{\left(r+B_{//,n}\right)}^{2}+{\left(B_{a,n}\right)}^{2}}\right)\approx 2B_{⊥,n}/\left(\lambda r\right),$$

$\rho_{e}$ is the resolution in elevation, given by [12]:

(9) $$\rho_{e}\approx \lambda r/\left(2B_{⊥,total}\right).$$

Here, $B_{⊥,total}=2N\cdot B\sin\alpha \cdot \sin\theta$ is the total orthogonal baseline. The elevation range after elevation processing is $\left[-H_{max}/2,H_{max}/2\right]$, where the maximum ambiguity in elevation $H_{max}$ is:

(10) $$H_{max}\approx \lambda r/\left(2B\sin\alpha \cdot \sin\theta \right).$$

From Equations (3) and (7), it can be seen that the signal in $\left(r^{\prime },y^{\prime },0\right)$ occurs at $\left(r^{\prime },y^{\prime },-y^{\prime }\cot\alpha /\sin\theta \right)$ after elevation processing, which means the azimuth signal of the target will be located at different positions in elevation, i.e., the mainlobe and sidelobes along the azimuth of the focused target will separate in elevation. Moreover, if $\left|-y^{\prime }\cot\alpha /\sin\theta \right|>H_{max}/2$, the signal will be aliased in elevation; the aliased position is given by:

(11) $$s^{\prime }=-y^{\prime }\cot\alpha /\sin\theta -\left\lfloor -y^{\prime }\cot\alpha /\sin\theta /H_{\max}+0.5\right\rfloor \cdot H_{\max},$$

where ⌊x⌋ is the largest integer not larger than x.

**Figure 3:**
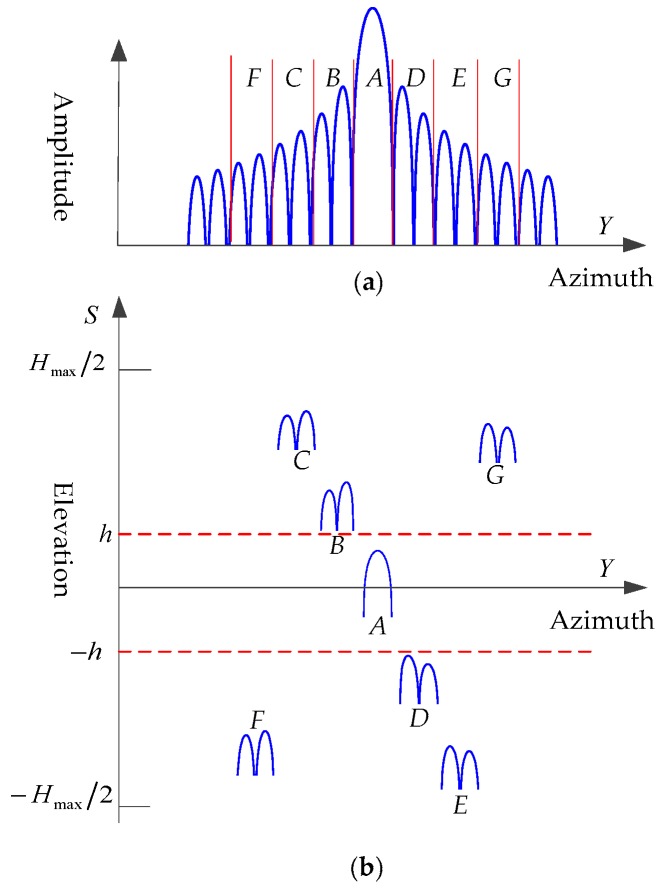
Effect of elevation processing. (**a**) Azimuth profile of a target in the 2D image; (**b**) slice across the 3D image in the azimuth-elevation plane.

To further explain the effect of elevation processing, Figure 3a shows the azimuth profiles of a target in a 2D image, and Figure 3b is a slice across the 3D image in the azimuth-elevation plane. It can be seen that after elevation processing, the sidelobes at different azimuth positions were shifted to different elevations. Moreover, the F and G parts in Figure 3a were located at the wrong positions due to aliasing in elevation, as given by Equation (11).

Thus, if we integrate the energy of the signal $ss\left(y^{\prime },r^{\prime },s\right)$ incoherently over the elevation range [−h,h] in which mainly the azimuth mainlobe and maybe several lower sidelobes are distributed, an image, $\tilde{s}(y^{\prime },r^{\prime })$, with low azimuth sidelobes is obtained:

(12) $$\tilde{s}\left(y^{\prime },r^{\prime }\right)={\int_{-h}^{h}\left|ss\left(y^{\prime },r^{\prime },s\right)\right|}^{2}ds.$$

It can be seen that the selection of h is crucial in determining the performance of the proposed sidelobes suppression method and it will be discussed below in detail.

## 4. Parameter Design

Optimization of the performance of the proposed method is based on three criteria:

### 4.1. The Energy of the Azimuth Mainlobe Must be Preserved

**Figure 4:**
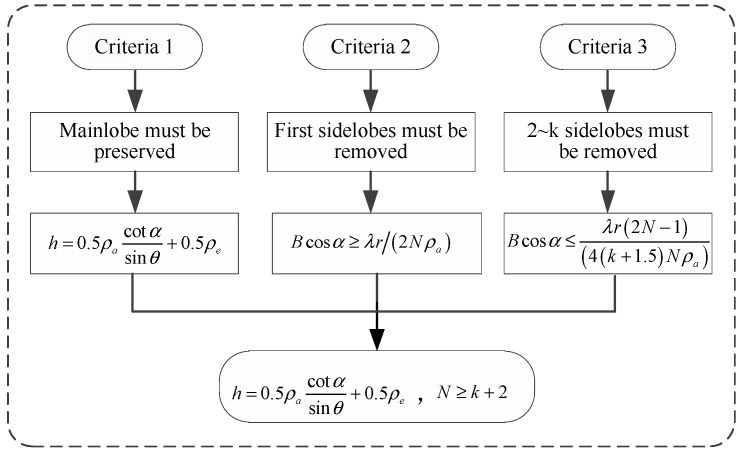
The design of parameter h.

From Equation (7), the azimuth signal is distributed in elevation. To preserve the energy of the azimuth mainlobe, the integrating range [−h,h] must contain the position of the azimuth mainlobe. This is distributed in space because a point target can occur anywhere within the resolution cell. We defined the azimuth mainlobe as the part of the azimuth profile (see Figure 4b) in which the energy exceeds −4 dB (where the maximum value of the azimuth profile is normalized to 0 dB). Setting

(13) $$10\log\left({\sin c}^{2}\left({y^{\prime }}_{0}/\rho_{a}\right)\right)=-4,$$

yields ${y^{\prime }}_{0}\approx 0.5\rho_{a}$. Assuming the center position of the azimuth mainlobe in elevation is $s_{0}$ (where $s_{0}\in \left(-0.5\rho_{e}\;,0.5\rho_{e}\right)$), the elevation range of the azimuth mainlobe is $\left[s_{0}-{y^{\prime }}_{0}\cot\alpha /\sin\theta ,\;s_{0}+{y^{\prime }}_{0}\cot\alpha /\sin\theta \right]$. So, to preserve the energy of the azimuth mainlobe of the target, we select,

(14) $$h={y^{\prime }}_{0}\cot\alpha /\sin\theta +0.5\rho_{e}=0.5\rho_{a}\cot\alpha /\sin\theta +0.5\rho_{e}.$$

### 4.2. The First Azimuth Sidelobes in Elevation Must Be Outside the Integrating Range [−h,h]

The zeroes of $\sin c\left(y^{\prime }/\rho_{a}\right)$ occur at the points,

(15) $${y^{\prime }}_{m}=\pm m\rho_{a}\left(m=1,2,\cdots \right).$$

The first zero point is ${y^{\prime }}_{1}=\rho_{a}$, so the following relationship must hold:

(16) $${y^{\prime }}_{1}\cot\alpha /\sin\theta -0.5\rho_{e}\geq h.$$

Inserting (9) and (14) into (16), we have:

(17) $${y^{\prime }}_{1}=\rho_{a}B\cos\alpha \geq \lambda r/\left(2N\rho_{a}\right).$$

### 4.3. The 2nd to the Kth Azimuth Sidelobes in Elevation Must Be Outside the Integrating Range [−h,h]

k is a number we select. With the increase of k, the performance of azimuth sidelobes suppression is better. Two cases need to be considered: (a) if the *k*th azimuth sidelobes is not aliased in elevation, it will be outside [−h,h] when (17) is satisfied, since ${y^{\prime }}_{k}\cot\alpha /\sin\theta -0.5\rho_{e}\geq {y^{\prime }}_{1}\cot\alpha /\sin\theta -0.5\rho_{e}$, where ${y^{\prime }}_{k}=k\rho_{a}$; (b) if the *k*th azimuth sidelobes are aliased in elevation, the following conditions must be satisfied:

(18) $$\{\begin{array}{l} {y^{\prime }}_{k+1}=\left(k+1\right)\rho_{a}\\ {y^{\prime }}_{k+1}\cot\alpha /\sin\theta +0.5\rho_{e}\leq H_{\max}-h \end{array},$$

Hence,

(19) $$B\cos\alpha \leq \lambda r\left(2N-1\right)/\left(4\left(k+1.5\right)N\rho_{a}\right).$$

The sidelobes beyond *k*th with lower energy will contribute little, whether they are relocated in or out the [−h,h] in elevation. 

The first to *k*th azimuth sidelobes up to and including the *k*th will be suppressed if (17) and (19) are met. Furthermore, based on (17) and (19), we have:

(20) N≥k+2,

which means that more flight passes allow more azimuth sidelobes to be removed. Figure 4 illustrates the process of the parameter selection.

In summary, the integrating range can be computed based on (14), and the baseline B, the flight angle α, and the number of passes 2N+1, can be optimized using (17), (19), and (20).

## 5. Performance Simulation

This section shows the performance based on simulations, using the parameters listed in Table 1 where PRF represents the pulse repetition frequency. The associated elevation resolution is about 55 m, and the maximum ambiguity in elevation (see (10)) is 1654 m. The integrating range in elevation is [−92 m,92 m]. Moreover, from (17) and (19), the first to the 10th azimuth sidelobes will be suppressed after incoherent addition.

### 5.1. Point Target Simulations

**Figure 5:**
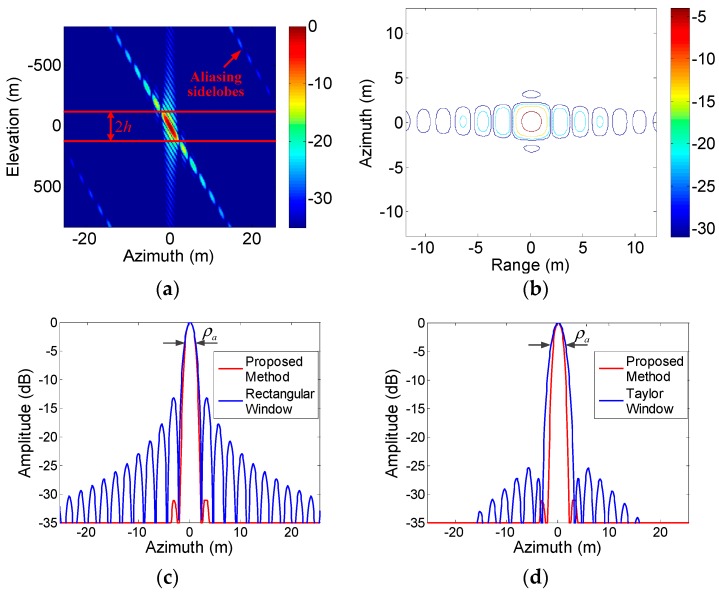
Imaging results for a point target. (**a**) Profile in the azimuth-elevation plane after elevation processing; (**b**) contour plots of the impulse response function (IRF) with the proposed method; (**c**) comparison of azimuth profiles using the proposed method (red) and the classical 2D focusing algorithm with a rectangular window (blue); (**d**) as for (**c**) but with a Taylor window (blue).

Simulations for a point target were first performed to test the proposed method, as shown in Figure 5. After elevation processing, a 3D image was obtained, and Figure 5a is a slice across this image in the azimuth-elevation plane. It can be seen that the azimuth sidelobes were compressed to different positions in elevation, and some sidelobes were aliased. The energy of the elevation signals between the two red lines was then incoherently integrated to form a 2D image of the impulse response function (IRF), as shown in the contour plot of Figure 5b. As can be seen, the target was well focused with low azimuth sidelobes. The azimuth profile obtained using the proposed method was compared with the (N+1)th 2D azimuth profile obtained by a classical 2D focusing algorithm, using a rectangular window (Figure 5c) and a Taylor window with parameters 0.25 (Figure 5d). The proposed method is seen to suppress azimuth sidelobes without degrading the azimuth resolution. The first sidelobes were preserved partly because they were in the integrating region, as shown in Figure 5a, but were less than −30 dB. This was much lower than that for the rectangular window (Figure 5c), and was achieved without the loss of resolution suffered when using the Taylor window (Figure 5d).

To further illustrate the performance of the proposed method, azimuth resolution, peak sidelobe ratio (PSLR), and integrated sidelobe ratio (ISLR) [17] were given as follows. As shown in Table 2, the azimuth resolution using the proposed method was 7.04% slightly higher than that for the rectangular window, at about 1.85 m. PSLR of the images weighted by rectangular window and Taylor window were −13.28 dB and −25.41 dB. ISLR of these two images were −10.11 dB and −20.18 dB, correspondingly. Through processing with the proposed method, the PSLR and ISLR of the image reached −31.07 dB and −29.36 dB, respectively, which were much lower than the other two images. Overall, it could be concluded that the proposed method can suppress azimuth sidelobes splendidly with the maintained azimuth resolution.

**Figure 6:**
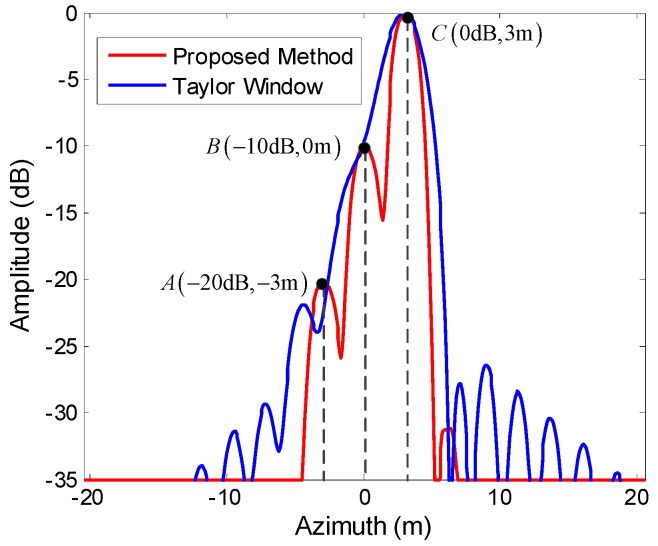
Azimuth profiles of three point targets using the proposed method and the classical 2D focusing algorithm.

Simulations with three point targets with different RCSs illustrate the advantages of the proposed method for detecting weak scatterers. The targets A, B, and C were located at −3 m, 0 m, and 3 m along the azimuth, with RCS −20, −10, and 0 dB, respectively, and they had the same height and range positions. Figure 6 compared the azimuth profiles using the proposed method and the classical 2D focusing algorithm with a Taylor window. The dashed lines in Figure 6 indicated the true target positions. It could be seen that they were clearly separated and had the correct RCS when the proposed algorithm was used. In contrast, under the classical 2D focusing algorithm, the weaker scatterers (A and B) were seriously affected by the sidelobes of the strong scatterer C and could not be detected. Moreover, the mainlobe of C was widened and affected by the sidelobes of B.

**Figure 7:**
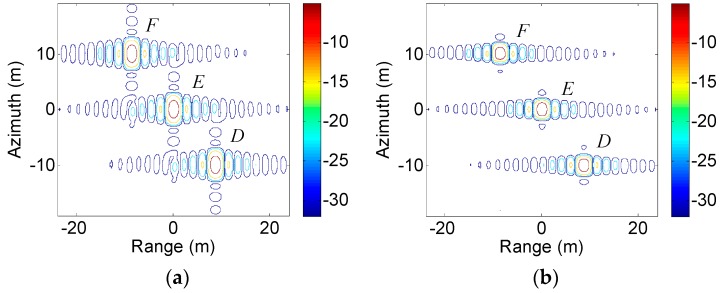
Contour plots of a scene with three point targets using: (**a**) classical 2D focusing algorithm with a Taylor window; (**b**) the proposed method.

Furthermore, simulations with three point targets at different heights were performed to verify the proposed method in a scene with height variations. The targets D, E, and F had the same range position and RCS and were located at (−10 m, −10 m), (0 m, 0 m), and (10 m, 10 m), where (*y*, *z*) are the (azimuth, height) coordinates. Figure 7a,b showed the contour plots of the IRF using the classical 2D focusing algorithm with a Taylor window in azimuth and the proposed method. It could be seen that the targets were focused at different range positions due to their different heights. The targets in Figure 7b were all well focused, and had lower azimuth sidelobes and better azimuth resolutions than those in Figure 7a.

### 5.2. Real SAR Image Simulations

Simulations were also performed on a TerraSAR-X image of a part of Dingxing airport, China, which contains 500 × 300 (azimuth × range) pixels. The original image from TerraSAR-X was used as RCS of the extended target to simulate MPS SAR data with the imaging geometry in Figure 1 and the simulation parameters listed in Table 1. It should be noted that the height of the targets in this real SAR scene is less than the elevation resolution 55 m. The focused 2D images were then processed by the proposed method to suppress the azimuth sidelobes.

**Figure 8:**
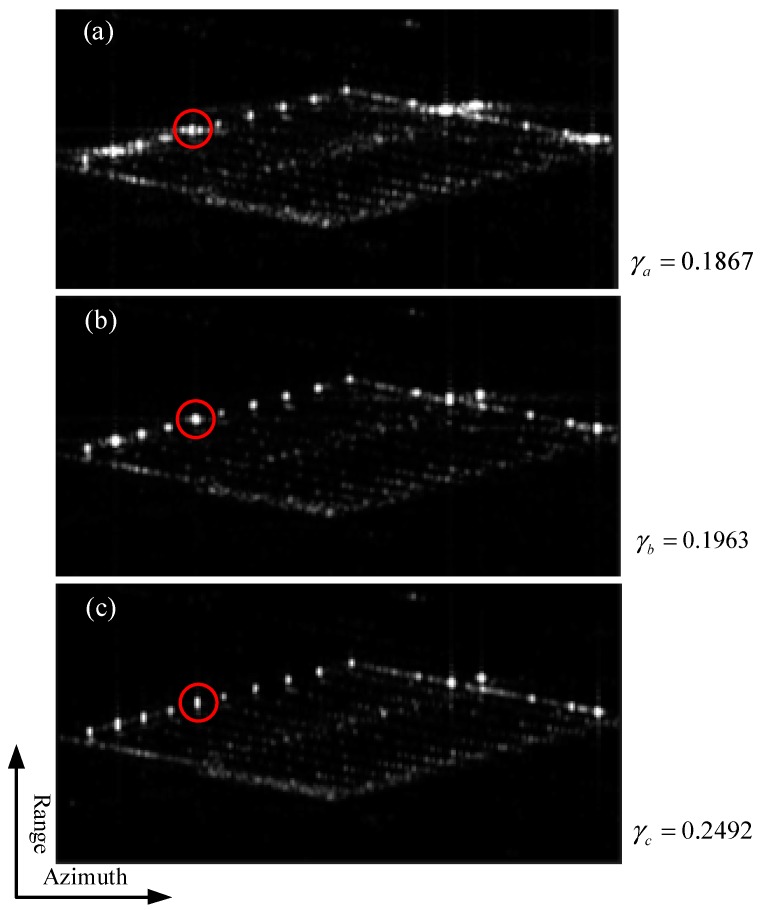
2D images obtained by processing the raw data simulated with a TerraSAR-X image of part of Dingxing airport, China, with: (**a**) classical 2D focusing algorithm with a rectangular window; (**b**) classical 2D focusing algorithm with a Taylor window; (**c**) the proposed method. The copyright of the original SAR image belongs to Airbus.

Figure 8 showed the resulting images, which were normalized to the same total energy. Figure 8a, b were formed using the classical 2D focusing algorithm with a rectangular window and a Taylor window in azimuth, respectively. High azimuth sidelobes can be seen in Figure 8a, which can be suppressed with a Taylor window at the expense of resolution (Figure 8b). Figure 8c was the image obtained using the proposed method. The targets now had low azimuth sidelobes and the mainlobes had not been widened, compared with those in Figure 8a,b (for example, see the target in the red circle).

As a measure of image focusing quality we used image contrast, γ, defined as the ratio of standard deviation and mean of image intensity. Typically, a larger contrast means better image quality. The values of contrast in the images in Figure 8 were $\gamma_{a}=0.1867$, $\gamma_{b}=0.1963$, and $\gamma_{c}=0.2492$, respectively; as expected, the image in Figure 8c had the highest contrast.

## 6. Conclusions

This article proposed a novel MPS SAR mode and a method to process MPS SAR data together with parameter selection criteria that can be used to optimize system design. Based on the MPS SAR mode, this is a novel application to suppress azimuth sidelobes using some of the existing algorithms, which were already adjusted to meet the new mode. Simulations indicated that it provided 2D images with lower azimuth sidelobes compared with some existing azimuth suppression methods. The analysis presented here is idealized, since it assumes flight passes whose center positions are collinear and equally spaced, which would in practice be difficult to satisfy. Future work will analyze the effects of relaxing these conditions.

## Figures and Tables

**Table 1:** List of simulation parameters.

Parameters	Value	Parameters	Value
Aircraft Height	20 km	Bandwidth	80 MHz
Incidence Angle	30°	Sample Rate	100 MHz
Wavelength	0.03 m	PRF	70 Hz
Velocity	100 m/s	Antenna Length	4.0 m
Flight Angle	2°	Pulse Duration	10
Baseline	12 m	Flight passes	31

**Table 2:** Imaging quality indicators for a single point target along azimuth.

Single Point Target	Resolution	PSLR	ISLR
Rectangular Window	1.99 m	−13.28 dB	−10.11 dB
Taylor Window	2.49 m	−25.41 dB	−20.18 dB
Proposed Method	1.85 m	−31.07 dB	−29.36 dB
